# Administration of Intravenous Ascorbic Acid—Practical Considerations for Clinicians

**DOI:** 10.3390/nu11091994

**Published:** 2019-08-23

**Authors:** Scott E. Walker, John Iazzetta, Shirley Law, Salmaan Kanji, Brigitte Bolduc, François Lamontagne, Neill K.J. Adhikari

**Affiliations:** 1 Leslie Dan Faculty of Pharmacy, University of Toronto, Toronto, ON M5S 3M2, Canada; 2 Department of Pharmacy, Sunnybrook Health Sciences Centre, Toronto, ON M4N 3M5, Canada; 3 Department of Pharmacy, The Ottawa Hospital and The Ottawa Hospital Research Institute, Ottawa, ON K1H 8L6, Canada; 4 Department of Pharmacy, Centre intégré universitaire de santé et de services sociaux de l’Estrie-Centre hospitalier universitaire de Sherbrooke, Sherbrooke, QC J1H 5N4, Canada; 5 Department of Medicine, Université de Sherbrooke and Centre de Recherche du Centre intégré universitaire de santé et de services sociaux de l’Estrie-Centre hospitalier universitaire de Sherbrooke, Sherbrooke, QC J1H 5N4, Canada; 6 Department of Critical Care Medicine, Sunnybrook Health Sciences Centre and Interdepartmental Division of Critical Care Medicine, University of Toronto, Toronto, ON M4N 3M5, Canada

**Keywords:** ascorbic acid, drug stability, administration, sepsis

## Abstract

Emerging data suggest that intravenous ascorbic acid (AA) may be beneficial in patients with sepsis. Clinicians require data on stability of diluted AA for safe administration. We evaluated the stability of AA diluted in normal saline (NS) or 5% dextrose in water (D5W) solutions over 14 days at 25 °C and at 4 °C, protected from light, using concentrations of 37 mg/mL and 77 mg/mL (Sandoz) and 40 mg/mL and 92 mg/mL (Mylan). We also assessed stability of a 40 mg/mL solution (Mylan) at 25 °C exposed to light for 75 h. Concentrations were measured using liquid chromatographic separation with ultraviolet light detection on days 0, 0.33, 1, 1.33, 2, 3, 4, 7, 10 and 14. By day 14, solutions at 4 °C retained >97.72% of the initial concentration; at 25 °C, solutions retained >88.02% of the initial concentration, but visual changes were evident after day 2. Multiple linear regression demonstrated that study day and temperature (*p* < 0.001) but not solution type (*p* = 0.519), concentration (*p* = 0.677) or manufacturer (*p* = 0.808) were associated with the percentage remaining. At 75 h, degradation rates were similar in solutions protected from vs. exposed to light. In conclusion, AA solutions are stable for at least 14 days at 4 °C, with protection from light.

## 1. Introduction

Intravenous ascorbic acid (vitamin C) has emerged as a potential treatment for sepsis [1]. Assessing stability of intravenous ascorbic acid (AA) over time is important for the feasibility of ongoing randomized trials and also for other clinical applications, such as cancer care [2]. The expiry date of intravenous medications following reconstitution or dilution is often limited to approximately 24 h to minimize the risk of administering a contaminated product. However, when reconstitution and dilution are carried out in a sterile environment following internationally recognized standards, such as USP 797 (USA) and National Association of Pharmacy Regulatory Authorities (NAPRA) Guidelines (2016, Canada), it is permissible to extend the beyond use date of these products if physical and chemical stability are demonstrated [3]. Extending the beyond use date may reduce drug waste [4,5], thereby reducing pharmacy workload, the cost of clinical research and the impact of drug shortages.

Our interest in AA stability pertains to an ongoing randomized trial in sepsis [6], in which a dose of 50 mg/kg of AA is administered intravenously every 6 h for 96 h [6]. If this dose is diluted in a 50 mL minibag, a 50 kg patient receiving a single dose of 2.5 g would have a concentration of 36.8 mg/mL or 39.7 mg/mL (allowing for 8 mL of overfill and drug volume using the formulations of Sandoz Canada (Boucherville QC Canada) and Mylan Canada Inc. (Etobicoke ON Canada), respectively), while a 130 kg patient would receive a 6.5 g per dose, corresponding to a concentration of 77.4 mg/mL or 91.6 mg/mL (including overfill and drug volume for the Sandoz and Mylan formulations, respectively). This concentration range is expected to include the majority of patients, but available data only support stability for up to 4 days for the 50 mg/mL concentration [7].

The primary objective of this study was to evaluate the stability and physical compatibility of 37, 40, 77 and 92 mg/mL solutions of AA injection following dilution in 50 mL polyvinyl chloride (PVC) bags of either 0.9% normal saline (NS) or 5% dextrose in water (D5W) during storage at 4 °C or 25 °C, with protection from light for 14 days. A secondary objective was to evaluate the stability of a 40 mg/mL solution of AA injection stored at 25 °C without protection from light, over a 75 h period. 

## 2. Materials and Methods

### 2.1. Materials

Two formulations of intravenous AA were studied: a 250 mg/mL solution per 2 mL ampoule from Sandoz and a 500 mg/mL per 50 mL vial from Mylan. The Sandoz formulation [8] also contains 0.15% sodium metabisulfite, while the Mylan formulation [9] also contains 0.25 mg/mL of disodium edetate. Both manufacturers recommend dilution with either normal saline (NS) or D5W solution, protection from light and administration within 4 h of dilution [8,9].

### 2.2. Chromatographic Analysis 

We used a liquid chromatographic separation with ultraviolet light detection (LC–UV) method adapted and re-validated from established LC–UV protocols [10,11,12]. The LC system consisted of a solvent delivery pump (Model P4000; Thermo Separation Products; San Jose, CA, USA), which pumped a mixture of 5% methanol and 95% 0.05 M phosphoric acid (HPLC grade, cat # P3786; Sigma Aldrich, Oakville, ON, Canada) through a 15 cm × 4.6 mm reversed-phase 5 µm column (Zorbax SB-CN; Agilent Technologies Canada Inc., Mississauga, ON, Canada) at 1.0 mL/min. Three microliters of each prepared sample, quality control or standard was injected directly onto the LC column using an autoinjector (Ultra WISP 715; Waters Scientific, Toronto, ON, Canada) in duplicate.

The column effluent was monitored with a variable wavelength UV detector (UV 6000 Thermo Separation Products; San Jose, CA, USA). The signal from the detector at 246 nm was integrated and recorded with a chromatography data system (Chrom Quest, version 5.0, ThermoFisher Scientific Inc., Nepean, ON, Canada). The area under the AA peak at 246 nm was subjected to least squares linear regression and the actual AA concentration in each sample determined by interpolation from the standard curve.

### 2.3. Assay Validation 

We confirmed the specificity of our method by evaluating its ability to detect degraded AA following the addition of varying concentrations of sodium hypochlorite [13,14,15]. Ten milliliters of an 80 mg/mL solution of AA was prepared using AA injection (500 mg/mL; Mylan Institutional LLC; lot 170910; expiry August 2020). To this solution, 0.01 mL of varying strengths of sodium hypochlorite (0.05–0.5%) was added to 0.5 mL of the stock solution, diluted in 5 mL of distilled water, vortexed and chromatographed immediately. Chromatograms from all samples were inspected for the appearance of additional peaks, and the AA peak was compared between samples for changes in concentration, retention time and peak shape (electronic overlay and numerical calculation of tailing). UV spectral purity (220–798 nm, 6 nm bandwidth, deuterium lamp: UV6000, Thermo Separation Products, Fremont, CA) of the AA in a chromatogram of a degraded sample (13.77% remaining) produced by sodium hypochlorite was compared to the spectrum of the authentic undegraded sample of AA in water taken at time zero. These steps are necessary to ensure that the method is specific and capable of accurately reporting stability.

The accuracy and reproducibility of standard curves were then tested over five days, and system suitability criteria (theoretical plates, tailing and retention time) were developed to ensure consistent chromatographic performance, allowing for accurate and reproducible concentration reporting [16].

### 2.4. Stability Study

The evaluation of the Sandoz (250 mg/mL per 2-mL ampoule; Sandoz Canada Inc.; lot HW8852; expiry March 2020) and Mylan (500 mg/mL per 50 mL vial; Mylan Institutional LLC; lot 170910; expiry August 2020) formulations were completed sequentially. For each formulation, on study day zero, eight 50 mL NS PVC bags containing 2.5 g of ascorbic acid and eight 50 mL NS PVC bags containing 6.5 g of ascorbic acid were prepared. An additional eight bags of each formulation were prepared using 50 mL bags of D5W. As the result of overfill in PVC minibags and drug volume added, the estimated actual concentrations were 37.04 and 77.84 mg/mL with the Sandoz formulation and 40 and 92 mg/mL using the Mylan formulation. In each of these studies, half the bags were stored in a refrigerator (4 °C) and the other half were stored at room temperature (25 °C). All solutions were stored protected from light.

#### 2.4.1. Assay Schedule

Sampling occurred immediately following preparation of the solutions and at 0.33 (8 h), 1, 1.33 (32 h), 2, 3, 4, 7, 10 and 14 days. On each of 10 study times over the 14-day study period, 3 bags stored at both temperatures, concentration and diluent were assayed for ascorbic acid content. Samples of 0.10 mL with concentrations of 37 and 40 mg/mL were further diluted with 10 mL of distilled water, and 3 µL was chromatographed in duplicate. For concentrations of 77 and 92 mg/mL, 0.10 mL of the sample was further diluted with 25 mL of distilled water, and 3 µL was chromatographed in duplicate.

#### 2.4.2. Ascorbic Acid Analysis

On each study day, 0.1 mL of a 500 mg/mL solution of ascorbic acid (Mylan; lot 170910; expiry August 2020) was diluted to 100 mL of distilled water. This solution was further diluted to prepare standards with final concentrations of 0.375, 0.250, 0.125 and 0.0625 mg/mL. When combined with a blank, these standards served to construct a standard curve. Three quality control samples with ascorbic acid concentrations of 0.30, 0.20 and 0.1 mg/mL were also prepared from a separate stock solution. A 3 µL volume of each standard or quality control sample was chromatographed in duplicate without dilution. Intraday and interday errors were assessed by the coefficients of variation (CV) of the peak areas of both quality control samples and standards.

#### 2.4.3. Physical Compatibility

On each study day, samples drawn from a fourth PVC minibag were inspected visually for changes in colour and particulate matter against a white and black background using hexadecimal (HEX) defined standardized yellow and orange-yellow colour charts [17].

### 2.5. Evaluation of the Need to Protect from Light

An evaluation of the need to protect the solution from light was completed on a nominal 40 mg/mL AA solution (500 mg/mL per 50 mL vial; Mylan Institutional LLC; lot 170910; expiry August 2020) packaged in two 50 mL NS PVC minibags (Baxter). In addition, two clear 120 inch DEHP-free PVC administration sets (ICU Medical) were primed with 10 mL of the same solution. The minibags and primed administration sets were stored at 25 °C; one bag and administration set were exposed to room fluorescent light, and the other bag and administration set were protected from light for the duration of the study. The solutions were sampled 7 times over a 75 h period. Samples were analyzed using the method described above, according to details in Section 2.4.2.

### 2.6. Statistical Analysis

After determining the CV of the assay, a power calculation determined that duplicate injection had the ability to distinguish between concentrations which were different by at least two percent within each individual container [18,19]. Means were calculated for replicated analyses. Univariate analysis of variance and multiple linear regression (IBM SPSS Statistics Version 20, Release 20.0.0) was used to determine if there was an association between the observed percentage remaining and study day, concentration, diluent, temperature or manufacturer. The a priori cutoff for significance was set at 0.05. Chemical stability was calculated from the intersection of the lower limit of the 95% confidence interval of the observed linear regression determined slope (degradation rate) and the time to achieve 90% of the initial concentration (Microsoft Excel 2013).

## 3. Results

### 3.1. Accelerated Degradation and Assay Validation

**Figure 1:**
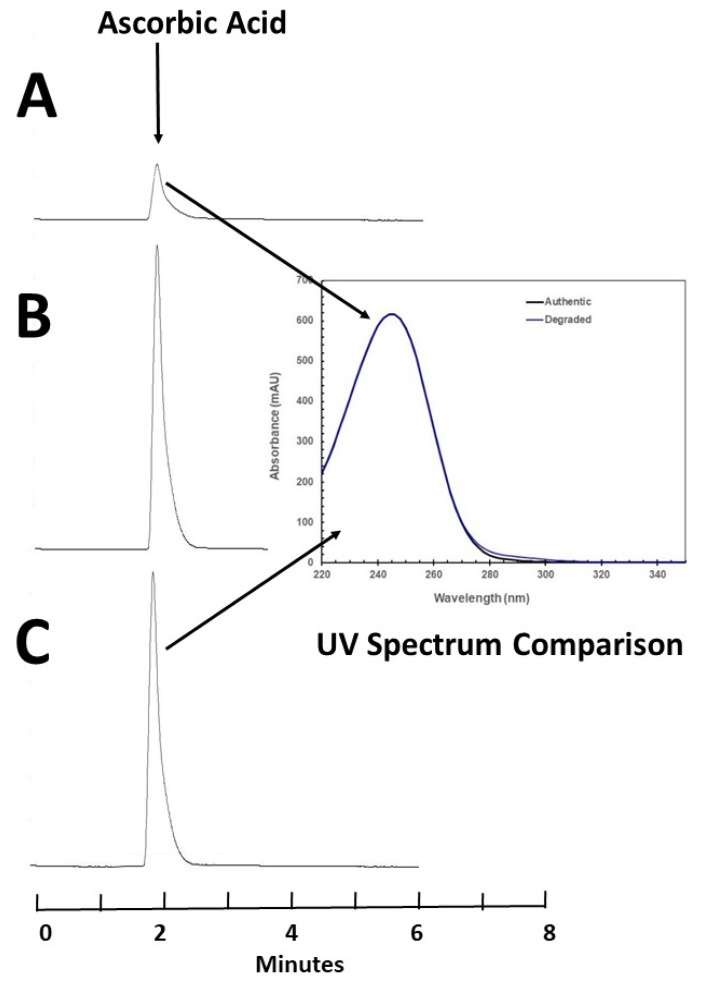
Chromatogram A represents an 80 mg/mL solution of ascorbic acid (AA) after the addition of sodium hypochlorite, which resulted in degradation of AA, leaving 13.8% of the initial concentration. No degradation products are observed. To confirm specificity, meaning the ‘purity’ of the AA peak, the UV spectrum was compared to the UV spectra observed in a fresh authentic (undegraded) AA peak (overlayed spectra, similarity 99.97%). Chromatogram C represents a 77 mg/mL solution in normal saline (NS) on study day zero. Chromatogram B represents the same 77 mg/mL solution in NS after 10 days storage at room temperature. The observed percentage remaining was 93.36%.

Degradation of AA with sodium hypochlorite occurred immediately and increased with increasing concentration of sodium hypochlorite. At 25 °C, an 80.0 mg/mL solution of AA in water was immediately degraded to 13.17% remaining when 10 μL of a 0.5% concentration of sodium hypochlorite was added to 0.5 mL of solution. Solutions containing lower concentrations of sodium hypochlorite degraded ascorbic acid to a lesser degree. When 10 μL of a 0.25% concentration of sodium hypochlorite was added, 41.15% remained when the sample was chromatographed immediately. Chromatography demonstrated that AA eluted at 2.0 min but failed to reveal degradation products (Figure 1). To confirm the specificity of the AA peak and demonstrate that degradation products did not interfere with the quantification of AA, the UV spectrum of the AA peak (220–798 nm) in a degraded sample was compared to the spectrum of the authentic undegraded standard and was found to be not different (similarity 99.97%, Figure 1), suggesting that the analytical method was specific for AA and can be judged as stability-indicating [13,14,15].

Analysis of standard curves and quality control samples during each study showed an average absolute deviation from the expected concentration of 2.12% and replicate error within a day averaged 0.24% and between days averaged 1.67%. During the studies, the standard deviation of regression, a measure of between-day analytical reproducibility, ranged from 0.33% to 0.81% (Sandoz formulation in Table 1 and Mylan formulation in Table 2). This finding indicates that differences of two per cent or more can be confidently detected within individual containers with acceptable error rates [18,19].

### 3.2. AA Stability

Concentrations observed on each study day for the Sandoz formulation and Mylan formulation are shown in Table 1 and Table 2, respectively. During the 14-day study period, all solutions retained more than 97.72% of the initial concentration when stored at 4 °C. Solutions stored at 25 °C retained more than 92.58% of the initial concentration for 10 days, but this decreased to 88.02% by day 14. The lower limit of the 95% confidence limit of concentration remaining was greater than 90 percent of the initial concentration, for all days sampled in the 14-day study period for solutions stored at 4 °C (Table 1 and Table 2). For solutions stored at room temperature, more than 90% of the initial concentration remained (based on the lower 95% confidence limit) for between 10.64 and 13.38 days.

All solutions stored at 4 °C and protected from light remained clear and colourless for the 14-day study period. All solutions stored at 25 °C and protected from light remained clear and colourless for 2 days before being observed as cream (HEX # FFFDDO) [17] on day 3. Based on observed concentrations in Table 1 and Table 2, this corresponds to a loss of more than 2% in the AA concentration. Colour of solutions stored at room temperature progressed to light mustard (concentrations of 37 and 40 mg/mL) or mustard (HEX # FFDB58) [17] for the higher concentrations (77 and 92 mg/mL) on day 14. No visible particles were observed in any solution during the study period.

All solutions stored at 4 °C and protected from light remained clear and colourless for the 14-day study period. All solutions stored at 25 °C and protected from light remained clear and colourless for 2 days before being observed as cream (HEX # FFFDDO) [17] on day 3. Based on observed concentrations in Table 1 and Table 2, this corresponds to a loss of more than 2% in the AA concentration. Colour of solutions stored at room temperature progressed to light mustard (concentrations of 37 and 40 mg/mL) or mustard (HEX # FFDB58) [17] for the higher concentrations (77 and 92 mg/mL) on day 14. No visible particles were observed in any solution during the study period.

Univariate analysis of variance was able to detect differences in percentage of AA remaining due to study day (*p* < 0.001) and temperature (*p* < 0.001), but not solution type (*p* = 0.457) or initial minibag concentration (*p* = 0.915). In this analysis, manufacturer and concentration were strongly correlated. Multiple linear regression was able to detect differences in percentage remaining of due to study day (*p* < 0.001) and temperature (*p* < 0.001), but not solution type (*p* = 0.519), concentration (*p* = 0.677) or manufacturer (0.808). 

### 3.3. Evaluation of the Need to Protect from Light

During the 75 h evaluation period, AA exposed to fluorescent room light at 25 °C nominally degraded faster than samples protected from light (Table 3). AA stored in the administration sets degraded to 95.15% remaining when exposed to light, compared to 96.79% remaining when protected from light (*p* = 0.948 for difference in degradation rate to 75 h). Similar rates of degradation were observed in PVC minibags (*p* = 0.681 for difference in degradation rate). 

## 4. Discussion

This study demonstrates that AA injection diluted in either NS or D5W retains more than 97.72% of the initial concentration over 14 days when stored at 4 °C. Degradation at room temperature is greater, but solutions retain at least 88.02% of original concentration at day 14, which corresponds to a degradation rate of 0.86% per day. Stability did not vary by manufacturer, suggesting that these products could be used interchangeably. Stability was also independent of concentration within the tested range of concentrations (37 mg/mL to 92 mg/mL). Visual changes did not occur over the study period at 4 °C, but at room temperature, the solutions changed colour by day 3 despite protection from light. Degradation of AA solutions stored in both minibags and clear PVC administration sets at room temperature did not significantly differ when protected from light vs. not, although degradation was nominally higher in the light-exposed samples. The implications of these findings for clinicians are that AA infusions (for example, a four-day course) can be prepared in advance, assuming intermittent bolus dosing, as long as the solutions are refrigerated, and the first dose of such solutions could be prepared in advance and stored in emergency departments or intensive care units. Protection from light during storage remains recommended, consistent with manufacturers’ advice, because we did not examine the full range of AA concentrations, storage temperatures or storage duration for this part of the study. Since the incremental rate of degradation during a 1 h AA infusion is considered negligible, protection from light of the minibag or infusion line during drug administration is unnecessary, although many clinicians would leave the opaque bag used to protect from light during storage in place for the infusion.

The colour changes by day 3 in room temperature samples may indicate AA degradation products, which we did not identify or measure, given that the peak we detected is specific for AA (Figure 1). The AA degradation pathway and products have been previously described [20,21,22]. Under aerobic conditions, the initial degradation product is dehydroascorbic acid, which is hydrolized to diketogulonic acid and further to threonic acid, oxalic acid and five-carbon compounds. Under anaerobic conditions, ascorbic acid is degraded to furfural. Oxalate is potentially nephrotoxic, and furfural is potentially carcinogenic. The unknown source of discolouration of AA solutions after 2 days of storage at room temperature should be investigated in additional studies, and until such data are available, administration of discoloured solutions to patients is strongly discouraged.

We identified 3 previous stability studies of ascorbic acid solutions in IV fluids [7,10,11]. Chin et al. [10] demonstrated the stability of a 9.03 mg/mL solution in NS and a 7.8 mg/mL solution in D5W at room temperature, protected from light for 24 h. Monti et al. [11] demonstrated the stability of a 75 mg/mL and a 100 mg/mL solution of ascorbic acid in sterile water, in the presence of either magnesium chloride or calcium gluconate, over 6 h at room temperature, after preparation. Carr et al. [7] demonstrated the stability of a nominal 30 mg/mL ascorbic acid solution in NS and a 50 mg/mL solution in D5W over 96 h, at 4 °C with protection from light or at ambient temperature and lighting. Accordingly, our results are consistent with previous AA stability experiments studies but also confirm stability at higher concentrations in both NS and D5W for a longer period of time (14 days at 4 °C and 10 days at room temperature (25 °C)), with protection from light. When assigning a before use date, in addition to chemical stability, other factors to consider should include sterility, risk level, storage conditions and compliance with national standards or guidelines governing the safe preparation of sterile products.

In light of the increasing demand for parenteral AA in both clinical and research settings, these findings will enable clinicians working in acute care settings to use IV solutions prepared in advance, while also reducing waste.

## 5. Conclusions

This study demonstrates that AA solutions at concentrations between 37 and 92 mg/mL, diluted in either NS or D5W and stored in PVC minibags, are physically, chemically and visually stable for at least 14 days refrigerated at 4 °C with protection from light. Clinicians in acute care settings may consider using AA solutions stored under these conditions. By contrast, when stored at room temperature (25 °C), AA solutions remained stable for 10 days, but discolouration was apparent after 2 days, possibly indicating degradation products of uncertain safety.

## Figures and Tables

**Table 1:** Ascorbic acid (Source: Sandoz) initial concentrations and percentage remaining ^1^ on each study day with calculation of the time to achieve 90% remaining (T-90) with 95% confidence.

Solution	NS	NS	NS	NS	D5W	D5W	D5W	D5W
Temperature	4 °C	25 °C	4 °C	25 °C	4 °C	25 °C	4 °C	25 °C
Nominal Concentration (mg/mL)	37 mg/mL	37 mg/mL	77 mg/mL	77 mg/mL	37 mg/mL	37 mg/mL	77 mg/mL	77 mg/mL
Actual Concentration (mg/mL)	36.64	36.71	77.42	77.21	36.85	36.87	77.21	77.21
Study Day								
0	100.00 ± 0.49	100.00 ± 0.81	100.00 ± 0.24	100.00 ± 0.13	100.00 ± 0.32	100.00 ± 0.13	100.00 ± 0.11	100.00 ± 0.18
0.33	99.66 ± 0.31	99.66 ± 0.52	99.27 ± 0.06	99.06 ± 0.24	99.30 ± 0.15	99.39 ± 0.11	98.62 ± 0.37	99.11 ± 0.16
1	99.23 ± 0.09	99.20 ± 0.46	99.68 ± 0.24	99.42 ± 0.13	98.92 ± 0.24	99.20 ± 0.20	99.58 ± 0.40	99.49 ± 0.38
1.33	98.56 ± 0.45	98.76 ± 0.57	99.26 ± 0.87	99.10 ± 0.18	98.79 ± 0.54	98.35 ± 0.25	99.54 ± 0.27	99.58 ± 0.33
2	98.77 ± 0.38	98.11 ± 0.44	98.89 ± 0.64	98.64 ± 0.54	98.60 ± 1.30	100.14 ± 1.02	99.00 ± 0.61	98.92 ± 0.14
3	98.62 ± 0.15	97.47 ± 0.82	98.74 ± 0.27	97.80 ± 0.54	99.16 ± 0.83	97.83 ± 0.10	99.03 ± 0.23	97.85 ± 0.22
4	98.74 ± 0.11	96.36 ± 0.80	97.72 ± 0.62	96.47 ± 0.36	98.85 ± 0.62	96.72 ± 0.28	99.06 ± 0.03	97.11 ± 0.14
7	98.44 ± 0.26	95.20 ± 0.83	97.74 ± 0.25	94.82 ± 0.47	97.98 ± 0.85	95.18 ± 0.27	98.83 ± 0.32	95.22 ± 0.19
10	99.09 ± 0.31	93.18 ± 0.74	98.36 ± 0.04	93.08 ± 1.08	99.11 ± 1.17	92.91 ± 0.71	99.39 ± 0.16	92.58 ± 1.01
14	98.91 ± 1.01	88.45 ± 0.81	98.56 ± 0.06	90.11 ± 0.20	99.32 ± 1.15	88.02 ± 0.28	98.94 ± 0.17	90.76 ± 0.45
Rate of change of concentration (%/day, slope) ^2^	−0.0368	−0.7625	−0.0965	−0.6929	−0.0146	−0.8068	−0.0265	−0.6837
Intercept	99.16	99.89	99.23	99.81	99.06	100.22	99.31	99.98
Correlation (r)	−0.3408	−0.9905	−0.5912	−0.9954	−0.1288	−0.9800	−0.2968	−0.9931
Standard Deviation of regression (Sy.x ) ^3^	0.501	0.524	0.650	0.330	0.556	0.808	0.421	0.398
Confidence Interval for slope ^4^	0.0828	0.0865	0.1073	0.0546	0.0919	0.1335	0.0696	0.0658
Fastest slope (%/day) ^4^	−0.1196	−0.8490	−0.2039	−0.7475	−0.1065	−0.9403	−0.0961	−0.7495
Slowest slope (%/day) ^4^	0.0460	−0.6760	0.0108	−0.6383	0.0772	−0.6732	0.0431	−0.6180
Shortest T−90 (days) ^5^	83.63	11.78	49.05	13.38	93.92	10.64	104.01	13.34

^1^ Concentrations are expressed as the percentage remaining ± standard deviation. Percentage remaining is calculated based on the concentration determined in duplicate of each of the 3 replicate vials stored at each temperature relative to the concentration on study day zero. ^2^ The degradation rate [slope] was determined by linear regression of the percentage remaining on each study day. ^3^ Sy.x is the standard deviation of regression. This is equivalent to the interday variability (error) of the analytical method, expressed as a percentage. ^4^ The confidence interval for the degradation rate allows calculation of the fastest and slowest degradation rate with 95% confidence (degradation rate ± confidence interval). ^5^ T-90 is the time in days for the concentration to decline by 10%, i.e., to achieve 90% of the initial concentration. The shortest T-90 uses the fastest degradation rate determined from the 95% confidence limit of the slope.

**Table 2:** Ascorbic acid (Source: Mylan) initial concentrations and percentage remaining ^1^ on each study day with calculation of the time to achieve 90% remaining (T-90) with 95% confidence.

Solution	NS	NS	NS	NS	D5W	D5W	D5W	D5W
Temperature	4 °C	25 °C	4 °C	25 °C	4 °C	25 °C	4 °C	25 °C
Nominal Concentration (mg/mL)	40	40	92	92	40	40	92	92
Actual Concentration (mg/mL)	39.45	39.48	91.25	91.23	39.30	39.54	91.38	91.26
Study Day								
0	100.00 ± 0.71	100.00 ± 0.46	100.00 ± 0.20	100.00 ± 0.22	100.00 ± 0.24	100.00 ± 0.57	100.00 ± 0.46	100.00 ± 0.42
0.33	100.02 ± 0.07	99.99 ± 0.20	99.90 ± 0.27	99.73 ± 0.18	99.95 ± 0.05	100.00 ± 0.23	100.13 ± 0.42	99.33 ± 0.72
1	99.07 ± 0.15	99.26 ± 0.09	99.13 ± 0.19	98.89 ± 0.12	99.27 ± 0.14	99.21 ± 0.36	99.34 ± 0.42	99.79 ± 0.59
1.33	98.82 ± 0.47	98.99 ± 0.15	98.98 ± 0.29	99.03 ± 0.11	99.28 ± 0.34	99.19 ± 0.44	99.08 ± 0.65	99.71 ± 0.32
2	98.94 ± 0.36	99.08 ± 0.29	99.08 ± 0.36	99.12 ± 0.14	99.50 ± 0.29	99.07 ± 0.61	99.24 ± 0.83	99.82 ± 0.38
3	98.20 ± 0.35	97.57 ± 0.52	98.95 ± 0.19	97.54 ± 0.50	99.04 ± 0.13	97.70 ± 0.16	99.44 ± 0.69	97.81 ± 0.38
4	99.06 ± 0.63	96.24 ± 0.75	98.62 ± 0.16	96.37 ± 1.00	99.05 ± 0.65	96.17 ± 0.74	99.16 ± 0.94	96.16 ± 0.17
7	98.77 ± 0.69	94.70 ± 0.32	98.08 ± 0.56	95.22 ± 0.35	98.82 ± 0.58	94.35 ± 0.57	99.17 ± 0.74	95.00 ± 0.53
10	99.49 ± 0.72	92.66 ± 0.42	98.77 ± 0.26	93.18 ± 0.42	99.54 ± 0.68	92.67 ± 0.74	99.86 ± 0.70	92.26 ± 0.98
14	99.09 ± 0.78	88.39 ± 0.77	98.02 ± 0.39	90.08 ± 0.57	98.99 ± 0.54	88.18 ± 1.26	98.87 ± 0.54	89.39 ± 0.73
Rate of change of concentration (%/day, slope) ^2^	−0.0198	−0.8110	−0.1106	−0.6932	−0.0430	−0.8288	−0.0359	−0.7850
Intercept	99.23	100.15	99.42	99.87	99.53	100.19	99.58	100.28
Correlation (r)	−0.1644	−0.9945	−0.7909	−0.9936	−0.4995	−0.9940	−0.3938	−0.9871
Standard Deviation of regression (Sy.x) ^3^	0.585	0.421	0.422	0.390	0.368	0.449	0.414	0.629
Confidence Interval for slope ^4^	0.0966	0.0695	0.0698	0.0644	0.0608	0.0741	0.0684	0.1038
Fastest slope (%/day) ^4^	−0.1164	−0.8805	−0.1804	−0.7576	−0.1039	−0.9029	−0.1043	−0.8889
Slowest slope (%/day) ^4^	0.0769	−0.7415	−0.0408	−0.6289	0.0178	−0.7546	0.0324	−0.6812
Shortest T−90 (days) ^5^	85.91	11.36	55.42	13.20	96.27	11.08	95.90	11.25

^1^ Concentrations are expressed as the percentage remaining ± the standard deviation. Percentage remaining is calculated based on the concentration determined in duplicate of each of the 3 replicate vials stored at each temperature relative to the concentration on study day zero. ^2^ The degradation rate [slope] was determined by linear regression of the percentage remaining on each study day. ^3^ Sy.x is the standard deviation of regression. This is equivalent to the interday variability (error) of the analytical method, expressed as a percentage. ^4^ The confidence interval for the degradation rate allows calculation of the fastest and slowest (upper) degradation rate with 95% confidence (degradation rate ± confidence interval). ^5^ T-90 is the time in days for the concentration to decline by 10%, i.e., to achieve 90% of the initial concentration. The shortest T-90 uses the fastest degradation rate determined from the 95% confidence limit of the slope.

**Table 3:** Effect of light on ascorbic acid concentrations and percentage remaining ^1^ at each sampling time with calculation of the time to achieve 90% remaining (T-90) with 95% confidence.

Container	Tubing	Tubing	Minibag	Minibag
Solution	NS	NS	NS	NS
Protected from Light/Light	Protected	Light	Protected	Light
Nominal Concentration (mg/mL)	40 mg/mL	40 mg/mL	40 mg/mL	40 mg/mL
Time (hours)\Actual Concentration (mg/mL)	39.58	39.18	39.58	39.18
0.5	100.03 ± 0.09	100.91 ± 0.22	99.65 ± 0.24	101.64 ± 0.90
1.0	99.88 ± 0.17	100.80 ± 0.01	99.72 ± 0.05	101.13 ± 0.08
2.0	99.94 ± 0.09	99.09 ± 0.08	99.17 ± 0.36	100.84 ± 0.01
6.0	99.62 ± 0.01	98.74 ± 0.04	99.70 ± 0.34	98.93 ± 0.08
24.0	99.00 ± 0.09	98.33 ± 0.05	99.05 ± 0.03	98.03 ± 0.00
75.0	96.79 ± 0.09	95.15 ± 0.03	96.63 ± 0.05	95.35 ± 0.27
Rate of change of concentration (%/day, slope) ^2^	−0.0425	−0.0666	−0.0405	−0.0736
Intercept	99.981	100.034	99.761	100.558
Correlation (r)	−0.999	−0.933	−0.972	−0.924
Standard Deviation of regression (Sy.x) ^3^	0.069	0.774	0.296	0.920
Confidence Interval for slope ^4^	0.00264	0.02948	0.01127	0.03501
Fastest slope (%/day) ^4^	−0.0451	−0.0961	−0.0518	−0.1086
Slowest slope (%/day) ^4^	−0.0399	−0.0371	−0.0293	−0.0386
Shortest T-90 (hours) ^5^	221.55	104.09	192.97	92.04

^1^ Concentrations are expressed as the percentage remaining ± the standard deviation. Percentage remaining is calculated based on the concentration determined in duplicate of each of the 3 replicate vials stored at each temperature relative to the concentration on study day zero. ^2^ The degradation rate [slope] was determined by linear regression of the percentage remaining on each study day. ^3^ Sy.x is the standard deviation of regression. This is equivalent to the interday variability (error) of the analytical method, expressed as a percentage. ^4^ The confidence interval for the degradation rate allows calculation of the fastest and slowest (upper) degradation rate with 95% confidence (degradation rate ± confidence interval). ^5^ T-90 is the time in hours for the concentration to decline by 10%, i.e., to achieve 90% of the initial concentration. The shortest T-90 uses the fastest degradation rate determined from the 95% confidence limit of the slope.
